# Duodeno-pancreatic and extrahepatic biliary tree trauma: WSES-AAST guidelines

**DOI:** 10.1186/s13017-019-0278-6

**Published:** 2019-12-11

**Authors:** Federico Coccolini, Leslie Kobayashi, Yoram Kluger, Ernest E. Moore, Luca Ansaloni, Walt Biffl, Ari Leppaniemi, Goran Augustin, Viktor Reva, Imitiaz Wani, Andrew Kirkpatrick, Fikri Abu-Zidan, Enrico Cicuttin, Gustavo Pereira Fraga, Carlos Ordonez, Emmanuil Pikoulis, Maria Grazia Sibilla, Ron Maier, Yosuke Matsumura, Peter T. Masiakos, Vladimir Khokha, Alain Chichom Mefire, Rao Ivatury, Francesco Favi, Vassil Manchev, Massimo Sartelli, Fernando Machado, Junichi Matsumoto, Massimo Chiarugi, Catherine Arvieux, Fausto Catena, Raul Coimbra, WSES-AAST Expert Panel, Offir Ben-Ishay, Matti Tolonen, Riccardo Bertelli, Tal Horer, Paula Ferrada, Isidoro Di Carlo, Bruno M. Pereira, Dario Parini, Giulia Montori, Belinda De Simone, Osvaldo Chiara, Andreas Hecker, Nicola DeAngelis, Carlos Augusto Gomes, Joseph Galante, Miklosh Bala, Konstantinos S. Mylonas, Anastasia Pikoulis, Paola Perfetti, Mircea Chirica, Joaquin Bado, Kenji Inaba, Neil Parry, Oreste Romeo, Martijn Stommel, Mohan Rajashekar, Edward Tan, Francesco Salvetti, Boris Sakakushev

**Affiliations:** 1 General, Emergency and Trauma Surgery Department, Pisa University Hospital, Via Paradisa, 2, 56124 Pisa, Italy; 2 Division of Trauma, Surgical Critical Care, Burns and Acute Care Surgery, University of California San Diego, San Diego, USA; 3 Division of General Surgery, Rambam Health Care Campus, Haifa, Israel; 4 Trauma Surgery, Denver Health, Denver, CO USA; 5 General, Emergency and Trauma Surgery Department, Bufalini Hospital, Cesena, Italy; 6 Trauma Surgery Department, Scripps Memorial Hospital, La Jolla, CA USA; 7 General Surgery Department, Mehilati Hospital, Helsinki, Finland; 8 Department of Surgery, Zagreb University Hospital Centre and School of Medicine, University of Zagreb, Zagreb, Croatia; 9 General and Emergency Surgery, Sergei Kirov Military Academy, Saint Petersburg, Russia; 10 Department of Surgery, DHS Hospitals, Srinagar, Kashmir India; 11 General, Acute Care, Abdominal Wall Reconstruction, and Trauma Surgery, Foothills Medical Centre, Calgary, Alberta Canada; 12 Department of Surgery, College of Medicine and Health Sciences, UAE University, Al-Ain, United Arab Emirates; 13 Trauma/Acute Care Surgery & Surgical Critical Care, University of Campinas, Campinas, Brazil; 14 Trauma and Acute Care Surgery, Fundacion Valle del Lili, Cali, Colombia; 15 3rd Department of Surgery, Attiko Hospital, National & Kapodistrian University of Athens, Athens, Greece; 16 Department of Surgery, Harborview Medical Centre, Seattle, USA; 17 Department of Emergency and Critical Care Medicine, Chiba University Hospital, Chiba, Japan; 18 Pediatric Trauma Service, Massachusetts General Hospital, Boston, MA USA; 19 General Surgery Department, Mozir City Hospital, Mazyr, Belarus; 20 Department of Surgery and Obstetrics and Gynecology, University of Buea, Buea, Cameroon; 21 General and Trauma Surgery, Virginia Commonwealth University, Richmond, VA USA; 22 General and Trauma Surgery Department, Pietermaritzburg Hospital, Pietermaritzburg, South Africa; 23 General and Emergency Surgery, Macerata Hospital, Macerata, Italy; 24 General and Emergency Surgery Department, Montevideo Hospital, Montevideo, Uruguay; 25 Department of Emergency and Critical Care Medicine, Saint-Marianna University School of Medicine, Kawasaki, Japan; 26 Clin. Univ. de Chirurgie Digestive et de l’Urgence, CHUGA-CHU Grenoble Alpes, UGA-Université Grenoble Alpes, Grenoble, France; 27 Emergency and Trauma Surgery, Maggiore Hospital, Parma, Italy; 28 Department of General Surgery, Riverside University Health System Medical Center, Moreno Valley, CA USA

**Keywords:** Pancreas, Bile duct, Biliary tree, Ampulla, Duodenum, Trauma, Adult, Pediatric, Classification, Guidelines, Injury, Surgery, Operative, Non-operative, Conservative, Endoscopic retrograde cholangiopancreatography (ERCP), Endoscopy

## Abstract

Duodeno-pancreatic and extrahepatic biliary tree injuries are rare in both adult and pediatric trauma patients, and due to their anatomical location, associated injuries are very common. Mortality is primarily related to associated injuries, but morbidity remains high even in isolated injuries. Optimal management of duodeno-bilio-pancreatic injuries is dictated primarily by hemodynamic stability, clinical presentation, and grade of injury. Endoscopic and percutaneous interventions have increased the ability to non-operatively manage these injuries. Late diagnosis and treatment are both associated to increased morbidity and mortality. Sequelae of late presentations of pancreatic injury and complications of severe pancreatic trauma are also increasingly addressed endoscopically and with interventional radiology procedures. However, for moderate and severe extrahepatic biliary and severe duodeno-pancreatic injuries, immediate operative intervention is preferred as associated injuries are frequent and commonly present with hemodynamic instability or peritonitis. The aim of this paper is to present the World Society of Emergency Surgery (WSES) and American Association for the Surgery of Trauma (AAST) duodenal, pancreatic, and extrahepatic biliary tree trauma management guidelines.

## Background

Duodeno-pancreatic and extrahepatic biliary tree injuries are, by definition, transitional lesions that may involve one or more anatomical structures. Their management is multidisciplinary. The initial phase is best managed by trauma or emergency surgeons but the late reconstructive phase should involve hepatobiliary surgeons. Moreover, endoscopy, interventional radiology, and gastroenterology may be involved to improve success of non-operative management (NOM) and to manage early and late sequelae of injury and complications. Transition of treatment strategies should occur as quickly and seamlessly as possible as morbidity and mortality both increase with delays in treatment.

Adult duodenal trauma has an incidence of 0.2–0.6% of all trauma patients and 1–4.7% of all cases of abdominal trauma [1–3]. Pediatric duodenal trauma is also rare, occurring in < 1% of all pediatric trauma and 2–10% of children with abdominal trauma [4–6]. Associated injuries are present in 68–86.5% of patients, with major vascular injury occurring in 23–40% of cases. Presence and type of associated injuries greatly impact the treatment of duodenal trauma [1, 2, 7–12]. Penetrating trauma is the most common cause of duodenal injury (DI) in adult patients, accounting for 53.6–90% of cases [2, 8–10, 12, 13]. Pediatric DI is most frequently due to blunt trauma which occurs in 70–78% of cases. Non-accidental trauma, motor vehicle crashes, and bicycle/handle bar injuries are the most common causes of pediatric DI [4–6]. Male gender is more commonly affected in both adult and pediatric DI.

Adult pancreatic injury (PI) is rare, occurring in less than 1% of all traumas and 3.7–11% of abdominal trauma [1–7]. Pediatric PI is also rare occurring in < 1% of children [8, 9]. Blunt trauma is the most common cause among both adults and children accounting for 61.1–89% of cases in most series, with motor vehicle and bicycle crashes being the most frequent causes [5, 6, 10–16]. However, penetrating mechanisms are much more common in studies from South Africa, North America, and the military [2–4]. Associated injuries are frequent, occurring in 55–100% of cases, and are more common in patients requiring surgery and following penetrating mechanisms of injury [1, 3, 6, 11, 12, 14, 17]. Male gender is more commonly affected, accounting for 63–79% of adults and 57–73% of pediatric PI [3, 5, 6, 8, 10–12, 14–16].

Extrahepatic biliary tree injury (EHBTI) is even rarer than pancreatic injury. EHBTI occurs in 0.1% of adult and 0.009% of pediatric trauma. Isolated EHBTI is extremely rare occurring in only 2–3% of cases [18–21]. The most frequently associated injuries include the liver, pancreas, and duodenum. Blunt trauma is more common than penetrating for all EHBTI except the gallbladder, which is more frequently injured due to penetrating mechanisms [18, 21, 22]. Management of EHBTI in both adults and children is primarily dictated by associated injuries and injury grade. The majority of EHBTI will require surgical or endoscopic management.

### Notes on the use of the guidelines

The guidelines are evidence-based, with the grade of recommendation based on the evidence. The guidelines present the diagnostic and therapeutic methods for optimal management of duodenal-bilio-pancreatic trauma. The practice guidelines promulgated in this work does not represent a standard of practice. They are suggested plans of care, based on best available evidence and the consensus of experts, but they do not exclude other approaches as being within the standard of practice. For example, they should not be used to compel adherence to a given method of medical management, which method should be finally determined after taking account of the conditions at the relevant medical institution (staff levels, experience, equipment, etc.) and the characteristics of the individual patient. However, responsibility for the results of treatment rests with those who are directly engaged therein, and not with the consensus group.

## Methods

**Fig. 1:**
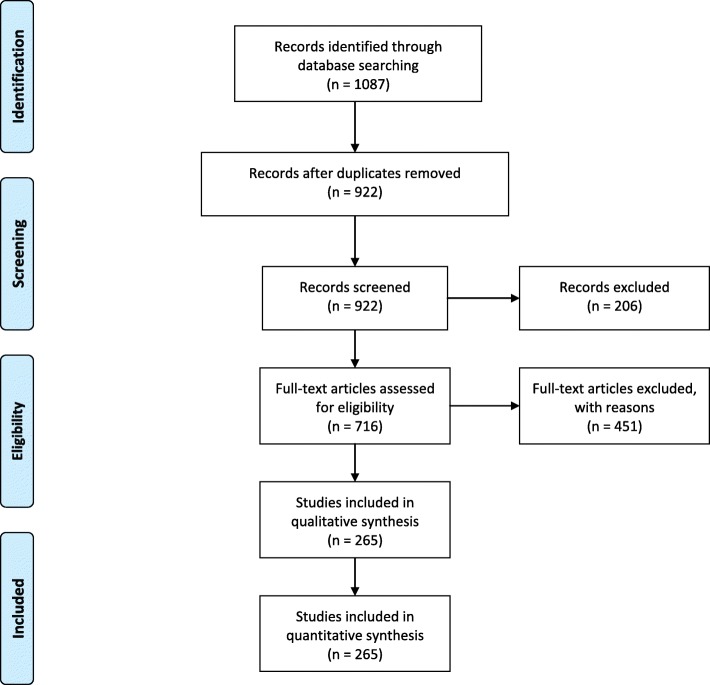
PRISMA flow chart

A computerized search was done by the bibliographer in different databanks (MEDLINE, Scopus, EMBASE). Citations were included for the period between January 1990 and March 2019 using the primary search strategy: duodenum, pancreas, bile duct, biliary tree, ampulla, trauma, adult, paediatric, classification, guidelines, injury, surgery, diagnosis, follow-up, operative, non-operative, conservative, endoscopic retrograde cholangiopancreatography (ERCP), endoscopic, management, combined with AND/OR. No search restrictions were imposed. The dates were selected to allow comprehensively published abstracts of clinical trials, consensus conference, comparative studies, congresses, guidelines, government publication, multicenter studies, systematic reviews, meta-analysis, large case series, original articles, and randomized controlled trials. Research details are summarized in Fig. 1. The level of evidence (LE) was evaluated using the GRADE system (Table 1) [23]. A group of experts in the field coordinated by a central coordinator was contacted to express their evidence-based opinion on several issues about the pediatric (< 16 years old) and adult duodeno-pancreatic and extrahepatic biliary tree trauma. Through the Delphi process, the different issues were discussed in subsequent rounds. The central coordinator assembled the different answers derived from each round. Each version was then revised and improved. The definitive version was discussed during the World Society of Emergency Surgery (WSES) World Congress held in June 2019 in Njimengen, The Netherlands, by a combined WSES-American Association for the Surgery for Trauma (AAST) expert group. The final version in which the agreement was reached resulted in the present manuscript. Statements are summarized in Table 2.

**Table 1:** GRADE system to evaluate the level of evidence and recommendation

Grade of recommendation	Clarity of risk/benefit	Quality of supporting evidence	Implications
1A			
Strong recommendation, high-quality evidence	Benefits clearly outweigh risk and burdens, or vice versa	RCTs without important limitations or overwhelming evidence from observational studies	Strong recommendation, applies to most patients in most circumstances without reservation
1B			
Strong recommendation, moderate-quality evidence	Benefits clearly outweigh risk and burdens, or vice versa	RCTs with important limitations (inconsistent results, methodological flaws, indirect analyses, or imprecise conclusions) or exceptionally strong evidence from observational studies	Strong recommendation, applies to most patients in most circumstances without reservation
1C			
Strong recommendation, low-quality or very low-quality evidence	Benefits clearly outweigh risk and burdens, or vice versa	Observational studies or case series	Strong recommendation but subject to change when higher quality evidence becomes available
2A			
Weak recommendation, high-quality evidence	Benefits closely balanced with risks and burden	RCTs without important limitations or overwhelming evidence from observational studies	Weak recommendation, best action may differ depending on the patient, treatment circumstances, or social values
2B			
Weak recommendation, moderate-quality evidence	Benefits closely balanced with risks and burden	RCTs with important limitations (inconsistent results, methodological flaws, indirect, or imprecise) or exceptionally strong evidence from observational studies	Weak recommendation, best action may differ depending on the patient, treatment circumstances, or social values
2C			
Weak recommendation, low-quality or very low-quality evidence	Uncertainty in the estimates of benefits, risks, and burden; benefits, risk, and burden may be closely balanced	Observational studies or case series	Very weak recommendation; alternative treatments may be equally reasonable and merit consideration

**Table 2:** Statement summary

	Statements
Diagnostic procedures	- Management of pediatric patients with duodenal-pancreatic trauma requires specific skills; only trauma centers should take care of this cohort of patients. (GoR 1C) - The choice of diagnostic technique at admission must be based on the hemodynamic status. (GoR 1A) - E-FAST is rapid, repeatable, and effective for detecting free fluid and solid organ injury. (GoR 1A) - Ultrasonography is not recommended to routinely diagnose duodeno-pancreatic trauma. Contrast-enhanced ultrasonography may have a diagnostic role in stable trauma patients with suspected pancreatic injury. (GoR 2B) - Repeated and combined measurement of serum amylase and lipase levels, starting from 3 to 6 h after the initial injury, is a useful tool to support clinical evaluation in suspicion of pancreatic injury. Elevated and/or increasing levels of serum amylase and lipase, in absence of definitive diagnosis, are indications for more accurate investigation. (GoR 1B) - Serial clinical examination is an important part of follow-up after biliary and pancreatic-duodenal trauma. (GoR 2A) - CT-scan with intravenous contrast is essential in diagnosing duodeno-pancreatic injuries in hemodynamically stable or stabilized trauma patients. (GoR 1A) - Administration of oral contrast material does not improve intravenous contrast-enhanced CT-scan sensitivity in detecting duodeno-pancreatic injuries. (GoR 2A) - A repeat CT-scan within 12–24 h from the initial injury should be considered in hemodynamically stable patients with high clinical suspicion for duodeno-pancreatic injury or pancreatic ductal injury with negative CT-scan or non-specific CT findings on admission imaging, and/or elevated amylase and lipase, or persistent abdominal pain. (GoR 2A) - Magnetic resonance cholangiopancreatography (MRCP) can be considered a second-line non-invasive diagnostic modality to definitely rule out pancreatic parenchymal and pancreatic ductal injuries. It should be considered for the diagnosis of suspected biliary injuries when performed with hepatobiliary contrast. (GoR 1B) - In pediatric patients and pregnant women, to detect pancreatic parenchymal or pancreatic duct lesions, MRI is preferred if available in the emergency setting. (GoR 2A) - In adult and pediatric patients, the risks associated with the radiation burden of CT should be balanced against the potential complications that may occur with a missed injury when alternative diagnostic modalities for pancreaticoduodenal injury are not available. (GoR 1C) - Abdominal plain films using water-soluble contrast in the early trauma scenario are not recommended. (GoR 2A) - Hepatobiliary scintigraphy is not recommended for detection of biliary leak in patients with suspected gallbladder and biliary injuries in the trauma setting. (GoR 2B) - Diagnostic peritoneal lavage does not improve the specificity of diagnosing duodeno-pancreatic injury. It is sensitive but not specific for biliary tract injury. (GoR 2B) - Exploratory laparotomy is indicated in hemodynamically unstable (WSES class IV) patients with a positive E-FAST. (GoR 1A) - During surgical exploration of patients with abdominal trauma, the duodeno-pancreatic complex must be exposed and explored. (GoR 1A) - During exploratory laparotomy, when biliary injury is suspected but not identified, an intraoperative cholangiogram is strongly recommended. (GoR 2A) - In patients who are clinically suspected of having duodenal-pancreatic injuries, and are deteriorating clinically, if the imaging is equivocal, a diagnostic laparotomy should be performed. (GoR 2A) - In suspected pancreatic duct and extrahepatic biliary tree injuries in hemodynamically stable or stabilized adults and pediatric patients, endoscopic retrograde cholangiopancreatography (ERCP) can be used for both diagnosis and treatment even in the early phase after trauma. (GoR 1B)
Non-operative management (NOM)	- Hemodynamic stability is the key factor in determining management strategy. (GoR 1C)
Duodenum	- Hemodynamically unstable (WSES class IV) patients should not be considered for NOM. (GoR 1C) - NOM can be considered for hemodynamically stable or stabilized patients with duodenal wall hematomas (WSES class I–II, AAST-OIS grade I–II) in the absence of other abdominal organ injuries requiring surgery. (GoR 2B) - Patients with progressive symptoms or worsening findings on repeat imaging should be considered failures of NOM. (GoR 2C) - Hematomas initially treated with NOM should be considered for operative management if duodenal obstruction has not resolved within 14 days. (GoR 2C)
Pancreas, biliary tree	- NOM should be the treatment of choice for all hemodynamically stable or stabilized minor PI WSES class I (AAST grade I and some grade II) and gallbladder hematomas without perforation WSES class I (AAST grade I) in the absence of other abdominal injuries requiring surgery. (GoR 2C) - Location of WSES class II (AAST grade III) PI is the primary determinant of treatment modality in hemodynamically stable adult patients. (GoR 2C) - NOM may be considered only in selected hemodynamically stable or stabilized patients with WSES class II (AAST grade III) very proximal pancreatic body injuries in the absence of other abdominal injuries requiring surgery and only in higher level trauma centers; success of NOM may be increased with utilization of endoscopic and percutaneous interventions. (GoR 2C). - Optimal management of hemodynamically stable or stabilized patients with WSES class III (AAST grade IV) PI is controversial. NOM management augmented by endoscopic or percutaneous interventions may be used in selected patients. (GoR 2C) - NOM of WSES class III (AAST grade IV) injuries should be considered only in an environment that provides around the clock capability for patient intensive monitoring, an immediately available endoscopy and interventional radiology suite, OR, and only in patients with stable or stabilized hemodynamic and absence of other abdominal injuries requiring surgery (GoR 2A). - Sequelae of PI such as pancreatic fistulae and pseudocysts can frequently be addressed with image-guided percutaneous drain placement, endoscopic stenting, internal drainage, and endoscopic cyst-gastrostomy or cyst-jejunostomy. (GoR 2C)
Operative management (OM)	- Hemodynamically unstable (WSES class IV) patients and those with peritonitis or bowel evisceration or impalement should undergo immediate operative intervention. (GoR 1C)
Duodenum	- Damage control techniques should be considered in hemodynamically unstable patients with DI, particularly those with associated injuries and physiologic derangement. (GoR 2B) - Primary repair of DI should be considered whenever technically possible regardless of grade of injury. (GoR 2B) - Ancillary procedures such as pyloric exclusion with and without gastrojejunostomy and biliary diversion may be considered in WSES class III or higher DI (AAST grades III, IV, and V). (GoR 2C) - Lesions requiring pancreaticoduodenectomy (Whipple procedure) are often accompanied by severe associated injuries and shock. Damage control techniques and staged reconstruction in subsequent phases performed by experienced surgeons should be considered. (GoR 2c)
Pancreas, biliary tree	- In WSES class I (AAST grade I and some grade II) PI found during exploratory laparotomy, drainage may be considered (GoR 2B). - Patients with distal WSES class II (AAST grade III) PI should undergo OM. (GoR 2C) - Distal pancreatectomy (with or without splenectomy) is the procedure of choice for distal WSES class II (AAST grade III) PI. (GoR 2C) - Pancreatoduodenectomy may be needed in patients with destructive injuries of the duodenal-pancreatic complex. In such cases, the operation has better results when performed in a staged fashion. Pancreato-jejunostomy or pancreato-gastrostomy reconstructions are equally effective in selected cases performed by experienced surgeons. (GoR 2C) - In extrahepatic biliary tree WSES class I injuries (AAST grade I, II, and III) with laceration, perforation, or avulsion of the gallbladder, cholecystectomy is the treatment of choice. (GoR 1C) - EHBT injuries undergoing an initial damage control procedure may be drained with delayed reconstruction performed as a staged approach. (GoR 2B) - EHBT WSES class II–III (AAST grades IV and V) injuries should undergo reconstruction with hepaticojejunostomy or choledochojejunostomy if there is no associated vascular injury. (GoR 2C) - NOM failure of EHBT WSES class II–III (AAST grades IV and V) injuries, hepaticojejunostomy should be considered during reconstruction. (GoR 2C)
Short- and long-term follow-up	- After discharge, the necessity for follow-up imaging should be driven by clinical symptoms (i.e., onset of abdominal distention, tenderness, fever, vomiting, jaundice). (GoR 2B) - In adults, CT-scan is usually the first-line follow-up imaging tool for new-onset signs and symptoms. (GoR 2A) - In pregnant females, the MRCP should be considered the diagnostic modality of choice for new-onset signs and symptoms, wherever available. (GoR 2A) - In pediatric patients, ultrasound or contrast-enhanced US should be the diagnostic modality of choice for follow-up imaging. If cross-sectional imaging is required, MRI is preferred. (GoR 2A) - Given the complexity and variability of traumatic injuries, the need for and choice of follow-up imaging should be made using a multidisciplinary approach. (GoR 2B)

### Definitions

*In adults patients*, *hemodynamic instability* is considered the condition in which admission systolic blood pressure is < 90 mmHg with evidence of skin vasoconstriction (cool, clammy, decreased capillary refill), altered level of consciousness and/or shortness of breath, or > 90 mmHg but requiring bolus infusions/transfusions and/or vasopressor drugs and/or admission base excess (BE) > − 5 mmol/L and/or shock index > 1 and/or transfusion requirement of at least 4–6 U of packed red blood cells within the first 24 h. *Transient responder patients* (adult and pediatric) are those showing an initial response to adequate fluid resuscitation, but then subsequent signs of ongoing blood loss and perfusion deficits. These patients have an initial response to therapy but do not reach sufficient stabilization to undergo interventional radiology procedures or NOM.

*In pediatric patients*, *hemodynamic stability* is considered a systolic blood pressure of 90 mmHg plus twice the child’s age in years (the lower limit is inferior to 70 mmHg plus twice the child’s age in years, or inferior to 50 mmHg in some studies). An acceptable hemodynamic status in children is considered a positive response to fluid resuscitation: 3 boluses of 20 mL/kg of crystalloid replacement should be administered before blood replacement leading to heart rate reduction, cleared sensorium, return of peripheral pulses, normal skin color, increase in blood pressure and urinary output, and an increase in warmth of the skin in the extremities. Clinical judgment, however, is fundamental in evaluating children.

#### WSES classification

The WSES classification divides duodenum, pancreas, and extrahepatic biliary tree injuries into four classes considering the AAST-OIS classification (Tables 3, 4, and 5) and the hemodynamic status (the final grade of the lesion depends on the higher grade lesion among the duodenal, pancreatic, and extrahepatic biliary tree) (Table 6):

- Minor (WSES class I)
- Moderate (WSES class II)
- Severe (WSES classes III and IV)

**Table 3:**
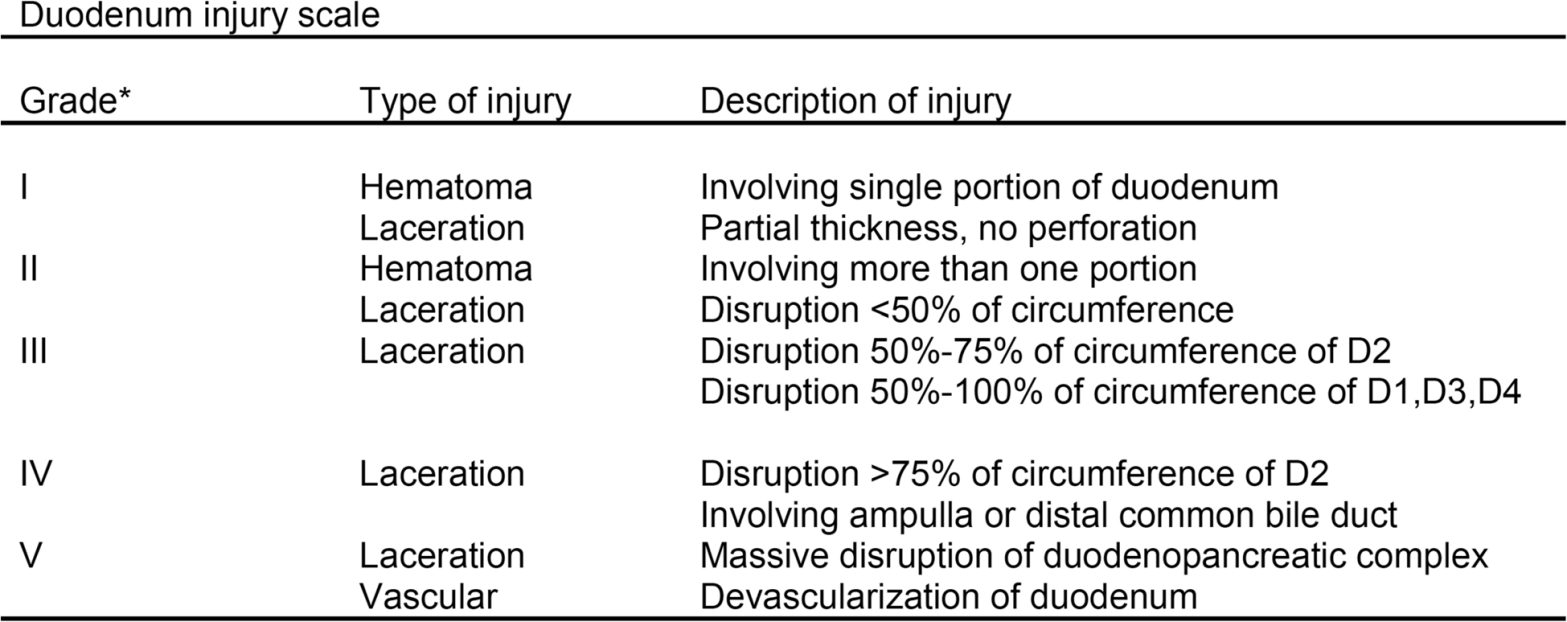
AAST organ injury scale for duodenal injuries

^*^Advances one grade for multiple injuries up to grade III. D1—first portion of duodenum; D2—second portion of duodenum; D3—third portion of duodenum; D4—fourth portion of duodenum)

**Table 4:**
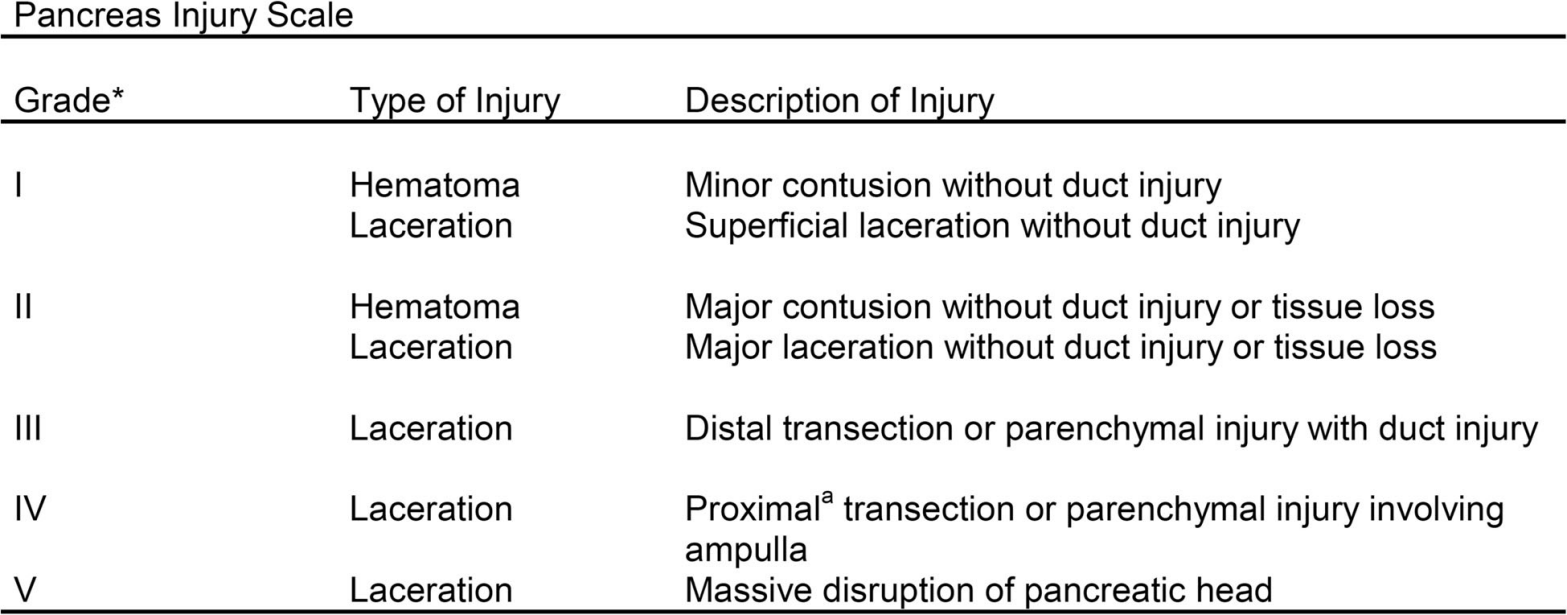
AAST organ injury scale for pancreatic injuries

^*^Advances one grade for multiple injuries up to grade III

^a^Proximal pancreas is to the patients’ right of the superior mesenteric vein

**Table 5:**
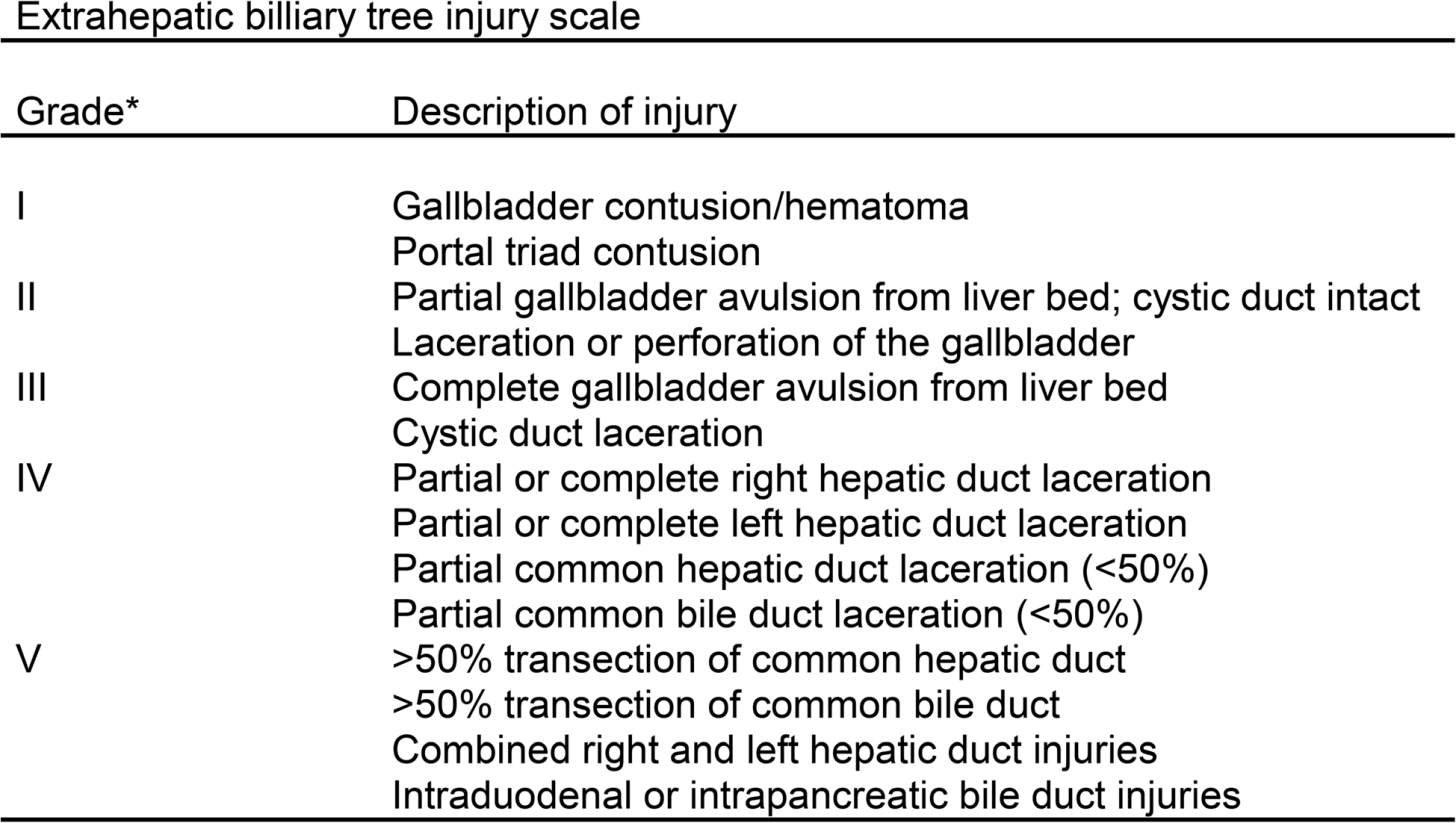
AAST organ injury scale for extrahepatic biliary tree injuries

^*^Advances one grade for multiple injuries up to grade III

**Table 6:** Duodeno-pancreatic and extra-hepatic biliary tree lesions

Grade	WSES class	Organ	AAST	Description of injury
Minor	WSES class I	Pancreas	I–II	- Minor contusion without duct injury Superficial laceration without duct injury - Major contusion without duct injury or tissue loss Major laceration without duct injury or tissue loss
		Duodenum	I	- Hematoma involving a single portion of duodenum Laceration: partial thickness, no perforation
		Extrahepatic biliary three	I–II–III	- Gallbladder contusion/hematoma. Portal triad contusion - Partial gallbladder avulsion from liver bed; cystic duct intact. Laceration or perforation of the gallbladder - Complete gallbladder avulsion from liver bed. Cystic duct laceration
Moderate	WSES class II	Pancreas	III	- Distal transection or parenchymal injury with duct injury
		Duodenum	II	- Hematoma involving more than one portion Laceration with disruption of less than 50% of circumference
		Extrahepatic biliary three	IV	Partial or complete right hepatic duct laceration Partial or complete left hepatic duct laceration Partial common hepatic duct laceration (< 50%) Partial common bile duct laceration (< 50%)
Severe	WSES class III	Pancreas	IV–V	- Proximal transection or parenchymal injury involving ampulla - Massive disruption of pancreatic head
		Duodenum	III–IV–V	- Disruption 50–75% of circumference of D2 Disruption 50–100% of circumference of D1, D3, and D4 - Disruption > 75% of circumference of D2 involving ampulla or distal common bile duct - Massive disruption of duodeno-pancreatic complex Devascularization of duodenum
		Extrahepatic biliary three	V	> 50% transection of common hepatic duct > 50% transection of common bile duct Combined right and left hepatic duct injuries Intraduodenal or intrapancreatic bile duct injuries
	WSES class IV	Any	Any	Any degree of lesion with hemodynamic instability

Minor duodeno-pancreatic and extrahepatic biliary tree injuries:

- WSES class I includes:
  - ◦ AAST-OIS grade I duodenal lesions
  - ◦ AAST-OIS grade I–II pancreatic lesions
  - ◦ AAST-OIS grade I–III extrahepatic biliary lesions

Moderate duodeno-pancreatic and extrahepatic biliary tree injuries:

- WSES class II includes:
  - ◦ AAST-OIS grade II duodenal lesions
  - ◦ AAST-OIS grade III pancreatic lesions
  - ◦ AAST-OIS grade IV extrahepatic biliary lesions

Severe duodeno-pancreatic and extrahepatic biliary tree injuries:

- WSES class III includes:
  - ◦ AAST-OIS grade III–IV–V duodenal lesions
  - ◦ AAST-OIS grade IV–V pancreatic lesions
  - ◦ AAST-OIS grade V extrahepatic biliary tree lesions
- *WSES class IV* includes hemodynamically unstable AAST-OIS grade I–V duodeno-bilio-pancreatic lesions

**Fig. 2:**
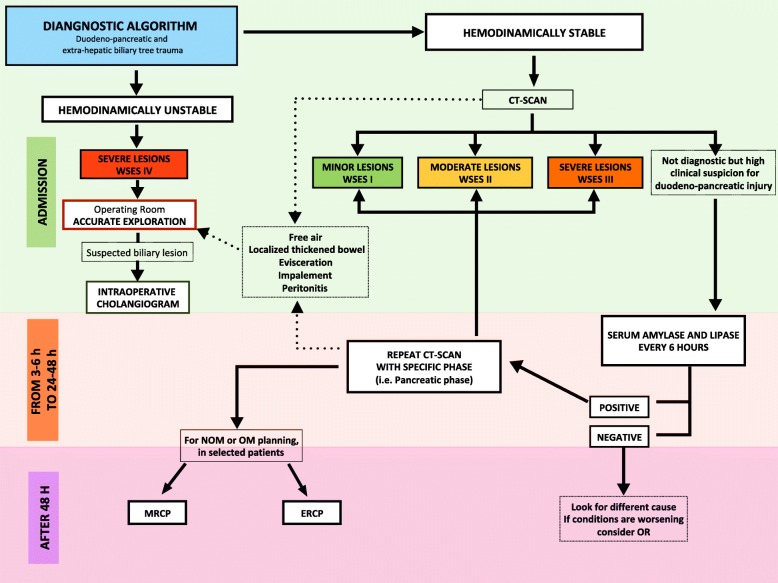
Diagnostic algorithm for duodeno-pancreatic and extrahepatic biliary tree traumatic lesions

**Fig. 3:**
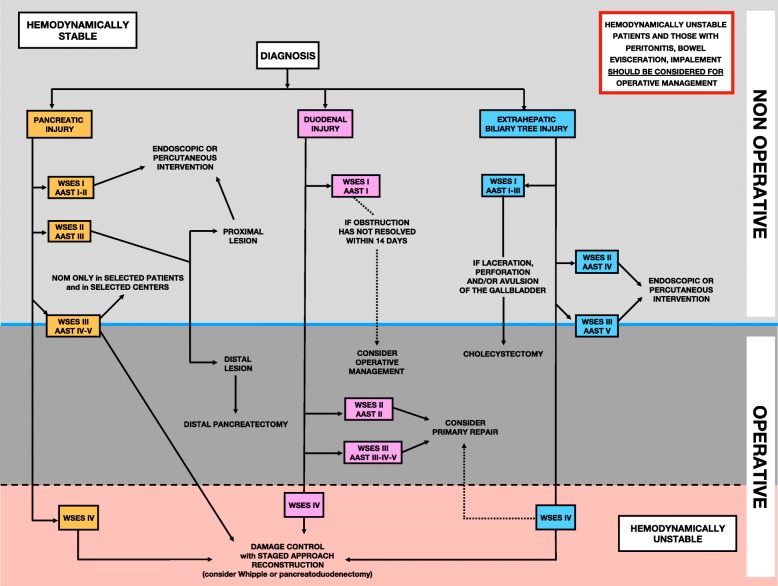
Management algorithm for duodeno-pancreatic and extrahepatic biliary tree traumatic lesions (asterisk indicates NOM should only be attempted in centers capable of a precise diagnosis of the severity of PI/DI/EHBTI and capable of intensive management (close clinical observation and hemodynamic monitoring in a high dependency/intensive care environment, including serial clinical examination and laboratory assay, with immediate access to diagnostics, interventional radiology, endoscopy, and surgery and immediately available access to blood and blood products)

Based on present classification, WSES and AAST suggest a diagnostic and management algorithm (Figs. 2 and 3, respectively).

### Diagnosis

- Management of pediatric patients with duodenal-pancreatic trauma requires specific skills; only trauma centers should take care of this cohort of patients. (GoR 1C)
- The choice of diagnostic technique at admission must be based on the hemodynamic status. (GoR 1A)
- Extended-Focused Assessment with Sonography for Trauma (E-FAST) is rapid, repeatable, and effective for detecting free fluid and solid organ injury. (GoR 1A)
- Ultrasonography is not recommended to routinely diagnose duodeno-pancreatic trauma. Contrast-enhanced ultrasonography may have a diagnostic role in stable trauma patients with suspected pancreatic injury. (GoR 2B)
- Repeated and combined measurement of serum amylase and lipase levels, starting from 3 to 6 h after the initial injury, is a useful tool to support clinical evaluation in suspicion of pancreatic injury. Elevated and/or increasing levels of serum amylase and lipase, in the absence of definitive diagnosis, are indications for more accurate investigation. (GoR 1B)
- Serial clinical examination is an important part of follow-up after biliary and pancreatic-duodenal trauma. (GoR 2A)
- CT-scan with intravenous contrast is essential in diagnosing duodeno-pancreatic injuries in hemodynamically stable or stabilized trauma patients. (GoR 1A)
- Administration of oral contrast material does not improve intravenous contrast-enhanced CT-scan sensitivity in detecting duodeno-pancreatic injuries. (GoR 2A)
- A repeat CT-scan within 12–24 h from the initial injury should be considered in hemodynamically stable patients with high clinical suspicion for duodeno-pancreatic injury or pancreatic ductal injury with negative CT-scan or non-specific CT findings on admission imaging, and/or elevated amylase and lipase, or persistent abdominal pain. (GoR 2A)
- Magnetic resonance cholangiopancreatography (MRCP) can be considered a second-line non-invasive diagnostic modality to definitely rule out pancreatic parenchymal and pancreatic ductal injuries. It should be considered for the diagnosis of suspected biliary injuries when performed with hepatobiliary contrast. (GoR 1B)
- In pediatric patients and pregnant women, to detect pancreatic parenchymal or pancreatic duct lesions, MRI is preferred if available in the emergency setting. (GoR 2A)
- In adult and pediatric patients, the risks associated with the radiation burden of CT should be balanced against the potential complications that may occur with a missed injury when alternative diagnostic modalities for pancreatico-duodenal injury are not available. (GoR 1C)
- Abdominal plain films using water-soluble contrast in the early trauma scenario are not recommended. (GoR 2A)
- Hepatobiliary scintigraphy is not recommended for detection of biliary leak in patients with suspected gallbladder and biliary injuries in the trauma setting. (GoR 2B)
- Diagnostic peritoneal lavage does not improve the specificity of diagnosing duodeno-pancreatic injury. It is sensitive but not specific for biliary tract injury. (GoR 2B)
- Exploratory laparotomy is indicated in hemodynamically unstable (WSES class IV) patients with a positive E-FAST. (GoR 1A)
- During surgical exploration of patients with abdominal trauma, the duodeno-pancreatic complex must be exposed and explored. (GoR 1A)
- During exploratory laparotomy, when biliary injury is suspected but not identified, an intraoperative cholangiogram is strongly recommended. (GoR 2A)
- In patients who are clinically suspected of having duodenal-pancreatic injuries, and are deteriorating clinically, if the imaging is equivocal, a diagnostic laparotomy should be performed. (GoR 2A)
- In suspected pancreatic duct and extrahepatic biliary tree injuries in hemodynamically stable or stabilized adults and pediatric patients, the endoscopic retrograde cholangiopancreatography (ERCP) can be used for both diagnosis and treatment even in the early phase after trauma. (GoR 1B)

The diagnosis of duodeno-pancreatic injuries represents a challenge. In blunt trauma, evidence of direct impact on the upper abdomen such as lower rib fractures, soft tissue ecchymosis, supra-umbilical seat belt sign, and upper lumbar spine fractures following a motor vehicle collision should suggest the involvement of the pancreas and duodenum. Penetrating trauma of the front side or back side of both the lower torso or upper abdomen should be considered highly suspicious for duodeno-pancreatic or extrahepatic biliary tree lesions if the diagnoses have not been ruled out by other diagnostic means.

Clinical signs of traumatic DI are highly non-specific, especially in the early post-traumatic period. Patients usually present with epigastric, right upper quadrant, or back pain 6–24 h after the injury, but the onset of pain has been reported as late as 5 days after injury [24, 25]. The most common test is the analysis of serum amylase and lipase [26]. However, in small-bowel injuries, initial amylase value does not differentiate between patients with perforated and non-perforated DI [27]. A normal amylase level does not exclude DI [28].

Persistently elevated or a rising level of amylase and lipase may be of prognostic significance for both pancreatic and duodenal injuries; therefore, measuring amylase and lipase levels every 6 h is recommended [29, 30]. Accuracy may be improved if they are measured more than 3 h after injury [31, 32].

On E-FAST, the presence of free fluid in the absence of solid organ injury may be a sign of hollow viscus injury; however, it has limited role in diagnosing acute pancreatic or duodenal injuries [28, 33, 34].

Serum amylase levels are neither sensitive nor specific for definitive screening or diagnosis of PI, particularly within 3–6 h after injury. Serum lipase is more specific than amylase [35–37]; serum lipase may support targeted screening of patients with clinical suspicion of PI [10, 11, 16, 35, 37–71].

Amylase is normal at admittance in up to 40% of patients with pancreatic trauma, and elevated levels are not specific for pancreatic trauma. Amylase can also be elevated in head, hepatic, and bowel injuries [61] and in alcohol abuse and after hypo-perfusion of the pancreas [26]. Lipase levels drawn on admission can be useful to exclude pancreatic injury but not to guide further investigation: negative predictive value (NPV) of normal lipase is 99.8%, but with positive predictive value (PPV) of 3.3% [36]. Amylase and lipase in association can reach sensitivity of 85% and specificity of 100%, with PPV of 100% and NPV of 96% (after 6 h from injury) [26, 69, 72]. Decreasing enzyme levels have been correlated with predicting success of NOM [16, 26, 35, 37, 40, 61, 70, 73]. Sensitivity of 88% and 96% NPV can be reached when amylase and lipase are associated to ultrasonography (US) [26, 36]. In low-resource settings, amylase and lipase, in combination with US, can be considered cost-effective methods to risk-stratify patients [26]. Persistently, elevated serum amylase after 10 days from the initial injury should be monitored closely given the increased risk of pseudocyst formation in both adults and children [26, 40, 52, 63, 65, 70, 73–77].

Contrast-enhanced CT-scan is the fastest and most comprehensive technique for evaluating duodeno-pancreatic injuries [78–80]. In duodenal trauma, CT-scan has a sensitivity and specificity of 86% and 88%, respectively, in diagnosing blunt hollow viscus injury [81–83]. However, missed blunt DI rates up to 27% have been described [84]. Of those with missed DI, 83% had subtle CT findings on retrospective review [85]. Careful CT-scan interpretation with clinical correlation is mandatory to avoid delayed diagnosis and treatment with increased morbidity and mortality [28, 60, 61, 67, 79, 80, 82, 86–90]. In fact, isolated periduodenal fluid or hematoma visualized on admission abdominal CT-scan does not necessitate immediate exploration [83, 91–94]. Intraperitoneal or retroperitoneal extraluminal air is a relatively specific sign of bowel perforation seen in 20–55% of patients; however, it may not be visible immediately after a traumatic perforation [95].

In pancreatic trauma, contrast-enhanced CT-scan has high specificity (90–95%) but low sensitivity (52–54%) for ductal involvement. Up to 40% of PI can be missed or misdiagnosed on abdominal CT-scan obtained within 12 h of injury [96, 97]. PI becomes more evident 12–24 h after trauma [41, 67, 98]. A repeat CT-scan with curved multi-planar reconstruction and specific pancreatic phase (35–40 s from iodine contrast injection) can help in diagnosing pancreatic ductal (PD) injuries [61, 67, 82]. Aggressive resuscitation or prolonged hypovolemia can produce radiological changes in pancreatic imaging; fluid overload can induce peripancreatic edema or collections. In patients with severe shock both hypo- and hyper-perfusion of the gland have been described [99–101].

A repeat CT-scan 12–48 h after admission in doubtful cases of pancreatic-duodenal lesions should be considered [91, 102]. The follow-up scan sensitivity for bowel perforation increases from 30 to 82% [103]. Moreover, the repeat CT-scan sensitivity for identification of an operative indication may increase up to 100% (67%). NPV for OM also increases from 94 to 100% with no increase in mortality or hospital length of stay [104, 105]. Complication rate is significantly higher only in those patients with delayed OM of more than 24 h [106].

The MRCP may be used in pancreatic-duodenal trauma to assess common bile duct/ampulla injury, and hepatobiliary contrast agents can help in localizing associated bile leaks. Minor injuries may be more evident on MRI than on CT-scan [79]. In association with secretin-dynamic study, the MRCP may diagnose pancreatic leakage [107, 108] and give additional information concerning parenchymal and proximal duct condition [71, 108, 109].

Oral contrast administration has not been shown to have substantial benefits in depicting bowel injuries when compared with CT-scan alone at the initial evaluation and during follow-up (sensitivity 95%, specificity 99.6%) [42, 102, 110–123].

Radiation-related risks in children and young patients must be considered. An increase in lifetime cancer-specific mortality of 801/4000 (20.00025%) to 800/4000 (20%) after CT-scan has been reported for American children [124]. However, the consequences of missed injury or delay in diagnosis on mortality and morbidity rates can be grave particularly with duodeno-pancreatic injuries.

Plain films of the abdomen are generally of little value in diagnosing duodeno-pancreatic injuries [125]; the same is true for upper gastrointestinal series using water-soluble contrast. Duodenography (oral contrast–enhanced fluoroscopic evaluation) for blunt and penetrating duodenal trauma in patients with equivocal CT-scan has an overall sensitivity of 25% for blunt DI and 54% for those requiring repair [126].

The ERCP may play a role in duodeno-pancreatic trauma in order to avoid late-diagnosis and/or treatment both in adult and pediatric patients [10, 15, 41–43, 48–52, 58–60, 62–64, 67, 68, 70, 76–78, 90, 97, 101, 127–149]. It is an invasive procedure with 3–14% risk of post-procedure pancreatitis and 0.2–1% mortality rate [6, 10, 11, 40, 41, 45, 49, 51–53, 58, 61–64, 67, 68, 70–72, 75, 77, 78, 97, 128, 130, 133, 134, 137–140, 142, 144, 146, 148–157]. Moreover, in suspected duodenal perforations, the ERCP is not recommended. Failed cannulation of the papilla of Vater or inadequate pancreatography can occur in up to 9–14% of patients [71, 137, 144, 152]. The small duct size in children is not an absolute contraindication for the ERCP in expert hands as it is relatively safe and effective [16, 53, 63, 64, 70, 76, 77, 134, 137, 139, 148, 152, 158]. Rates of PD cannulation may be influenced by duodenal mucosal edema and/or hematomas and anatomical changes [71]. Despite of these limitations, the ERCP may have a role in decreasing time from definitive diagnosis of duct injury and first treatment in selected cases [131, 159]. However, cross-sectional imaging should be performed before proceeding with the ERCP.

Hepatobiliary scintigraphy (HIDA) is not frequently used in the initial work-up of the acute trauma patient due to long scan times and limited resource availability [128].

Percutaneous transhepatic cholangiogram (PTC) could be considered after non-feasible or unsuccessful ERCP for diagnosis and treatment [21].

Diagnostic peritoneal lavage (DPL) has sensitivity higher than 99% for hemoperitoneum but it is neither specific nor reliable for the assessment of retroperitoneal injuries, with undetected bowel perforation seen in up to 10% of cases [160–163]. DPL alone is associated with a high number of unnecessary laparotomies [164], with consequent short- and long-term complications. Moreover, DPL is associated with a 0.8–2.3% risk of specific complications [165, 166].

Diagnostic laparoscopy has both diagnostic and therapeutic potentials in a delayed setting. Whenever negative, it may reduce the number of unnecessary laparotomies [167]. It has a growing role in the evaluation of penetrating abdominal trauma but it has not been specifically studied for the evaluation of pancreatic-duodenal injuries. The duodeno-pancreatic anatomy and the retroperitoneal location increase the risk of missed injuries [168]. Moreover, laparoscopy in trauma requires adequate training and experience as well as sufficient staffing and equipment [169, 170].

Ultimately, in the patient with diagnostic uncertainty and in the patient with persistent or worsening clinical signs and symptoms, radiologic and/or laboratory alterations due to an intra-abdominal lesion, laparotomy should be strongly considered [171]. For penetrating trauma, a thorough and meticulous exploratory laparotomy with retroperitoneal exposure and assessment remains critical in detecting pancreatic and duodenal injuries [172].

If exploration is negative but there is still a strong suspicion of DI, methylene blue administration through a naso-oro-gastric tube could be considered. During emergency laparotomy, the use of intraoperative pancreatography does not add to the visual findings [145]. Intraoperative cholangiogram through the cystic duct may help in defining EHBTI [87, 173]. Additional information can be provided by the use of intraoperative US of the pancreas; however, the lack of strong evidence and the necessity of trained surgeons make this technique not recommended or routinely used in trauma [130].

### Treatment

#### Non-operative management—duodenum

- Hemodynamic stability is the key factor in determining management strategy. (GoR 1C)
- Hemodynamically unstable (WSES class IV) patients should not be considered for NOM. (GoR 1C)
- NOM can be considered for hemodynamically stable or stabilized patients with duodenal wall hematomas (WSES class I–II, AAST-OIS grade I–II) in absence of other abdominal organ injuries requiring surgery. (GoR 2B)
- Patients with progressive symptoms or worsening findings on repeat imaging should be considered failures of NOM. (GoR 2C)
- Hematomas initially treated with NOM should be considered for operative management if duodenal obstruction has not resolved within 14 days. (GoR 2C)

#### Non-operative management—pancreatic and biliary tree

- NOM should be the treatment of choice for all hemodynamically stable or stabilized minor PI WSES class I (AAST grade I and some grade II) and gallbladder hematomas without perforation WSES class I (AAST grade I) in the absence of other abdominal injuries requiring surgery. (GoR 2C)
- Location of WSES class II (AAST grade III) PI is the primary determinant of treatment modality in hemodynamically stable adult patients. (GoR 2C)
- NOM may be considered only in selected hemodynamically stable or stabilized patients with WSES class II (AAST grade III) very proximal pancreatic body injuries in the absence of other abdominal injuries requiring surgery and only in higher level trauma centers; success of NOM may be increased with utilization of endoscopic and percutaneous interventions. (GoR 2C)
- Optimal management of hemodynamically stable or stabilized patients with WSES class III (AAST grade IV) PI is controversial. NOM management augmented by endoscopic or percutaneous interventions may be used in selected patients. (GoR 2C)
- NOM of WSES class III (AAST grade IV) injuries should be considered only in an environment that provides around the clock capability for patient intensive monitoring, an immediately available endoscopy and interventional radiology suite, OR, and only in patients with stable or stabilized hemodynamic and absence of other abdominal injuries requiring surgery. (GoR 2A)
- Sequelae of PI such as pancreatic fistulae and pseudocysts can frequently be addressed with image-guided percutaneous drain placement, endoscopic stenting, internal drainage, and endoscopic cyst-gastrostomy or cyst-jejunostomy. (GoR 2C)

NOM is similar between adult and pediatric patients and is dependent on hemodynamic stability, clinical presentation, and associated injuries. Shock is generally due to associated injuries, which are present in 55–100% of pancreatic-duodenal injuries, and are more frequent among patients with penetrating mechanism of injury [1, 3, 6, 7, 11, 12, 14, 17, 174–183].

Physical exam findings associated with DI are non-specific and may be more reliable in children. Serial observations may increase the sensitivity of physical exam findings in diagnosing DI [57, 184]. CT-scan is generally the standard of care in diagnosing DI. Patients with definite evidence of full thickness laceration such as extravasation of enteral contrast or free air should undergo immediate operative intervention. These findings are rare, and in the vast majority of patients, findings are either non-specific such as duodenal wall thickening, periduodenal edema, stranding, or free fluid, or they are entirely absent [62, 84, 91]. NOM should include serial abdominal exams, bowel rest, and nasogastric tube (NGT) decompression. Parenteral nutrition may be required if obstruction persists beyond 7 days [185]. Obstruction due to duodenal hematoma will generally resolve within 14 days; if not, operative decompression may be required [185–188]. Operative evacuation can be done open or laparoscopically [188]. Percutaneous drainage of duodenal hematomas is a viable alternative [185, 189–193].

NOM of duodenal hematomas is generally successful in both adults and children [62, 91, 105, 185, 194]. Failed NOM (fNOM) rates between 5 and 10.3% have been reported, with no differences in length of stay. In patients with fNOM, a 0–3% complication rate and reduced mortality compared with the group undergoing immediate OM has been reported [91, 105].

Minor PI is treated similarly in adults and children. Hemodynamically stable patients without associated operative injury should undergo a trial of NOM. Total parenteral nutrition (TPN) may be required in 62–73% of pediatric and 22.6% of adults [8, 12, 15, 16]. NOM of class I injuries is successful in 96–100% of pediatric and 80–92.2% of adults [6, 11, 15, 105, 195, 196] and is associated with reduced morbidity, mortality, and shorter length of stay [3, 105].

In WSES class II (AAST-OIS grade III) injuries in hemodynamically stable or stabilized patients, location of the injury largely determines optimal treatment. WSES class II injuries distal to the superior mesenteric vein (AAST-OIS grade III) should be managed operatively by resection with or without splenectomy as OM is associated with improved recovery times, and reduced morbidity in both adults and pediatrics [197–199]. Isolated proximal WSES class II and III injuries (AAST-OIS grade III and IV–V) may be considered for NOM. Although no randomized controlled trials exist, several large database studies and meta-analyses have demonstrated that NOM is pursued in 46% of pediatric and 28–48.5% of adult patients [3, 6, 15].

NOM of WSES moderate and severe PI (AAST-OIS grade III and IV–V) has been reported more among pediatric than adult patients with a success rate up to 89% [15]. NOM success rate in adults is about 30%. Pseudocyst rate was higher among NOM patients and in 65–74% of cases they were also managed non-operatively [15, 16]. Length of stay was similar between NOM and OM [9, 200].

Endoscopic and percutaneous interventions such as ERCP with pancreatic stent and/or sphincterotomy or percutaneous aspiration and drain placement for pancreatic duct injury have been reported in patients with class II and III PI (AAST-OIS grades III and IV–V) with success rates of 68–94% with or without the octreotide administration [15, 201–208]. However, some concerns exist regarding increased rates of pancreatic duct stricture [209].

Many EHBTI will be diagnosed at the time of laparotomy. However, in patients undergoing NOM, concern for EHBTI should prompt immediate investigation with MRCP or HIDA scan. Patients with gallbladder wall hematoma without perforation can be managed expectantly [18]. NOM can be attempted in hemodynamically stable patients with WSES grade II and III injuries (AAST-OIS grade IV–V) without definite indication for surgical intervention. In these cases, fluid collections should be drained percutaneously and the ERCP with stent placement should be attempted to address ductal lacerations. Very little data exist about NOM of EHBTI but a few small case series have demonstrated success in both adult and pediatric patients [18, 19, 21].

#### Operative management—duodenum

- Hemodynamically unstable (WSES class IV) patients and those with peritonitis or bowel evisceration or impalement should undergo immediate operative intervention. (GoR 1C)
- Damage control techniques should be considered in hemodynamically unstable patients with DI, particularly those with associated injuries and physiologic derangement. (GoR 2B)
- Primary repair of DI should be considered whenever technically possible regardless of grade of injury. (GoR 2B)
- Ancillary procedures such as pyloric exclusion with and without gastrojejunostomy and biliary diversion may be considered in WSES class III or higher DI (AAST grades III, IV, and V). (GoR 2C)
- Lesions requiring pancreaticoduodenectomy (Whipple procedure) are often accompanied by severe associated injuries and shock. Damage control techniques and staged reconstruction in subsequent phases performed by experienced surgeons should be considered. (GoR 2c)

#### Operative management—pancreas and biliary tree

- In WSES class I (AAST grade I and some grade II) PI found during exploratory laparotomy, drainage may be considered. (GoR 2B)
- Patients with distal WSES class II (AAST grade III) PI should undergo OM. (GoR 2C)
- Distal pancreatectomy (with or without splenectomy) is the procedure of choice for distal WSES class II (AAST grade III) PI. (GoR 2C)
- Pancreatoduodenectomy may be needed in patients with destructive injuries of the duodenal-pancreatic complex. In such cases, the operation has better results when performed in a staged fashion. Pancreato-jejunostomy or pancreato-gastrostomy reconstructions are equally effective in selected cases performed by experienced surgeons. (GoR 2C)
- In extrahepatic biliary tree WSES class I injuries (AAST grades I, II, and III) with laceration, perforation, or avulsion of the gallbladder, cholecystectomy is the treatment of choice. (GoR 1C)
- EHBT injuries undergoing an initial damage control procedure may be drained with delayed reconstruction performed as a staged approach. (GoR 2B)
- EHBT WSES class II–III (AAST grades IV and V) injuries should undergo reconstruction with hepaticojejunostomy or choledochojejunostomy if there is no associated vascular injury. (GoR 2C)
- NOM failure of EHBT WSES class II–III (AAST grades IV and V) injuries, hepaticojejunostomy should be considered during reconstruction. (GoR 2C)

Due to the high percentage of associated injuries in patients with duodeno-pancreatic and extrahepatic biliary three injuries, shock and peritonitis are common at or shortly after presentation. Hemodynamic instability is present in 10–44% of patients [210–215]. All patients with hemodynamic instability or peritonitis should proceed immediately to OM. Hemodynamically stable patients with CT findings of full thickness laceration, or class III DI (AAST-OIS grade III–IV–V), such as free air or extravasation of enteral contrast from the duodenum or an associated operative injury should also undergo immediate OM.

Damage control surgery (DCS) is reported in 20–63% of cases particularly in patients with associated vascular injuries and/or higher grade duodeno-pancreatic lesions. DCS has been associated with improved survival and equivalent or improved complication rates [2, 211, 212, 216, 217]. DCS is rarely needed for isolated DI, and the extent of the primary surgery will relate primarily to associated vascular injuries. Once hemostasis has been achieved, the DI can be addressed at the initial surgery if the patient’s physiology allows. The majority of DI found at laparotomy are WSES class I–II lacerations (AAST-OIS grade I–II). They should be repaired primarily in a tension-free transverse fashion after complete exposure and removal of all devitalized tissue. A nasogastric tube (NGT) should be placed to allow for proximal decompression. There is no evidence supporting routine periduodenal drain placement.

Management of WSES class III lacerations (AAST-OIS grade III–IV–V) not involving massive disruption of the duodeno-pancreatic complex is controversial. They are associated with a high mortality and high duodenal-specific morbidity (duodenal leak, fistula and anastomotic breakdown) with consequent abdominal sepsis and poor outcomes [218, 219]. Duodenal diverticulization and triple tube decompression are no longer advocated for the treatment of DI [187, 218, 219]. Most modern studies advocate primary repair, NGT decompression, and external drain placement even with large, high-grade injuries. In cases where primary repair is not possible, segmental resection and primary duodeno-duodenostomy could be performed. These more conservative techniques have demonstrated good outcomes with similar or better mortality and duodenal-related morbidity compared with more complex drainage and reconstructive procedures [57, 181, 194, 211–213, 216, 217]. Pyloric exclusion (PE) is still utilized although definite indications for it remains controversial [220]. Temporary PE has been described both with and without gastrojejunostomy. The pylorus can be stapled without transection or sutured internally with absorbable material so it will open spontaneously several weeks post-injury [221, 222], or sutures can be removed endoscopically. Several studies reported no improvement in morbidity, mortality, and a prolonged length of stay with PE compared with primary repair with NGT decompression alone [212, 214, 215, 217, 223, 224]. Moreover, concerns exist regarding the possible PE increasing the length of the procedure, complications, and risks of gastric suture line and marginal ulcers [105, 222, 224–226].

WSES class III injuries with massive disruption of the duodeno-pancreatic complex (AAST-OIS grade III–IV–V for duodenum and AAST-OIS grade IV–V for pancreas) are rare and require complex reconstruction. In the first or proximal second duodenal portion lesions where primary repair or resection and primary anastomosis are not possible, antrectomy and gastrojejunostomy with closure of the duodenum is an option [186]. In case of injuries located distal to the ampulla, a Roux-en-Y duodeno-jejunostomy can be performed [186, 187, 212]. When the ampulla or distal common bile duct is involved, re-implantation into healthy adjacent duodenum or reconstruction with a Roux-en-Y jejunal limb is an option if the adjacent tissue loss and injury are minimal [186]. When the duodenum and/or pancreatic head are severely devitalized or devascularized, pancreaticoduodenectomy (Whipple procedure) may be required. Associated injuries and severe physiologic derangements are common with these injuries [227–230]. DCS is required in 26–80% of cases and should be strongly considered at the time of initial operation [227–230]. It seems to improve survival and reduce complications in treating severe pancreatic-duodenal injuries requiring Whipple procedures [230]. Staged procedures have been suggested to improve outcomes. The assistance of experienced hepatobiliary surgeons should be defined on a case-by-case basis [187, 227–229]. Both classic Whipple procedures and pylorus preserving reconstructions are options dependent on the location of the DI and associated injuries [227, 231].

Delayed bowel function and obstruction from duodenal edema, hematoma, or stricture are common following DI [232]. To ensure adequate nutrition, a feeding jejunostomy may be considered in patients with severe duodeno-pancreatic injuries requiring resection and reconstruction; however, jejunostomy-related complications can occur in up to 7% of patients and intolerance to enteral nutrition is common [211, 232]. Total parenteral nutrition (TPN) may be required in 37–75% of patients [57, 185, 213].

Patients with PI who are hemodynamically unstable (33–50%) (WSES class IV) or have peritonitis should undergo immediate OM [1, 6, 14, 233]. Associated hollow viscus injury or operative intra-abdominal injury will be present in 24–82% of PI [4, 5, 11, 233]. DCS should be considered in patients with shock and exsanguinating hemorrhage. Surgical management of pancreatic injury is dependent on grade, location, and extent of associated injuries. Intraoperatively diagnosed WSES class I PI DCS (AAST-OIS grade I–II) can be managed expectantly, and closed suction drain placement is recommended for larger contusions and lacerations [234, 235]. Suture repair of lacerations should be avoided as it is associated with increased risk of pseudocyst formation [235]. WSES class II PI injuries (AAST-OIS grade III) involving the main pancreatic duct distal to the superior mesenteric vein (SMV) should be treated with distal pancreatectomy with or without splenectomy as OM is associated with improved recovery times, and reduced morbidity in both adult and pediatric PI [13, 197–199, 235]. Decreased incidence of pancreatic fistula when the pancreas was stapled rather than sewn has been demonstrated; however, ductal ligation made no difference [13]. Splenic preservation among trauma surgeons remains controversial. No significant increase in morbidity or mortality and a reduced length of stay associated with spleen preservation has been demonstrated [236]. Spleen preservation is of great importance in pediatric trauma patients; however, there is little data on splenic salvage in this cohort [237, 238]. Ultimately, the decision to preserve or remove the spleen will depend on the patient’s physiology, associated splenic injury, and the surgeon’s level of experience.

Optimal management of WSES class III PI (AAST-OIS grade IV–V) with transection of parenchyma/duct proximal to the SMV remains controversial. Subtotal and total pancreatectomy for proximal injuries may result in endocrine and exocrine dysfunction. Because of this, initial management includes debridement, oversewing the proximal pancreatic stump, and distal drainage with pancreaticojejunostomy (not well tolerated in physiologically deranged patients). These procedures are associated with high rates of pancreas-related (fistula) and overall complications. Modern studies predominantly utilize debridement and wide local drainage with good success [2, 4, 14, 239]. Drainage alone for proximal PI has rates of pancreatic fistula of 12–13.8% [238, 240] which compares favorably with small series of more complex reconstructions with pancreaticoenterostomy (11–20%) [241, 242].

WSES class III PI (AAST-OIS grade IV–V) with complete destruction or devascularization of the pancreatic head and pancreatico-duodenal complex is a specific and rare circumstance. Most of these patients require pancreaticoduodenectomy and present in shock and with severe associated injuries and should be treated with DCS [243]. Mortality after trauma Whipple remains high varying from 12 to 33%, but it may be improved with DCS techniques and appropriate patient selection [231, 244, 245]. Mortality with more conservative surgical treatments (duodenal reconstruction and drainage) appears to be similar, but complications, particularly pancreatic fistula, may be higher when compared with the Whipple procedure [13, 246].

Gallbladder WSES class I injuries (AAST-OIS grade I–II–III) account for approximately 30–60% of EHBTI [18–20]. The majority of these injuries are noted at the time of laparotomy. For all injuries except gallbladder wall hematomas, the treatment of choice is cholecystectomy [18, 19, 22]. Extrahepatic bile duct injuries often occur in conjunction with severe liver, duodenal, and pancreatic injuries. In these instances, management is dictated as much by the severity of the associated injuries as by the grade of the bile duct injury itself. In most cases, treatment of the injury with distal ligation and reconstruction with a Roux-en-Y hepaticojejunostomy is recommended [18, 19, 21]. Choledochojejunostomy may be used for distal common bile duct injuries in the absence of associated vascular injury that may compromise the blood supply to the anastomosis. Primary repair of WSES class II injuries (AAST-OIS grade IV) over a T-tube can be attempted but may result in strictures and need for future reconstructive surgery [18]. OM with Roux-en-Y hepaticojejunostomy is also recommended for patients with WSES class II and III injuries (AAST-OIS grade IV–V) after fNOM [18, 21].

### Follow-up

- After discharge, the necessity for follow-up imaging should be driven by clinical symptoms (i.e., onset of abdominal distention, tenderness, fever, vomiting, jaundice). (GoR 2B)
- In adults, CT-scan is usually the first-line follow-up imaging tool for new-onset signs and symptoms. (GoR 2A)
- In pregnant females, the MRCP should be considered the diagnostic modality of choice for new-onset signs and symptoms, wherever available. (GoR 2A)
- In pediatric patients, ultrasound or contrast-enhanced US should be the diagnostic modality of choice for follow-up imaging. If cross-sectional imaging is required, MRI is preferred. (GoR 2A)
- Given the complexity and variability of traumatic injuries, the need for and choice of follow-up imaging should be made using a multidisciplinary approach. (GoR 2B)

CT-scan is usually the first-line imaging tool in the assessment of late complications of pancreatic trauma and very useful in driving management [39, 61, 71, 72, 76, 96, 135, 145, 233, 247, 248]. MRI is a reliable alternative to CT-scan in children and pregnant women [40, 45, 52, 97, 249, 250].

US or CEUS is used as an alternative to CT for follow-up of fluid collections, pseudocysts, and pancreatic disruptions after pancreatic trauma mainly in children or in low-resource settings [16, 26, 40, 45, 49, 53, 55, 63, 71, 75, 78, 133, 134, 138, 245, 247, 251–254]. CEUS may improve results of pancreatic imaging, being nearly as accurate as CT-scan and reducing radiation exposure in children [249, 255, 256].

The ERCP is a useful tool in diagnosis, management, and follow-up of late complications such as pseudocysts, pancreatic fistulas (i.e., trans-papillary stenting), or main duct strictures secondary to injury or prolonged stenting (i.e., ERCP with pancreatic duct dilatation and stenting), even in pediatric patients [10, 39, 40, 45, 53, 67, 74, 137, 138, 148, 152, 154, 247, 253].

NOM of high-grade pancreatic lesions (WSES class III, AAST-OIS grade IV–V) requires stringent follow-up for at least 6 months to detect early and late sequelae [45].

### Complications

*Pseudocyst* is the most frequent complication following NOM [15, 52, 53, 64, 68, 69, 72, 154, 257, 258]. CT-scan is useful in evaluating pseudocysts and peripancreatic fluid collections following PI [96, 247, 259, 260] and in guiding percutaneous drainage [40]. US and endoscopic US (EUS) can also be used for follow-up and to guide percutaneous treatment of pseudocyst and abscess avoiding radiation exposure [45, 63, 70, 158, 247, 253]. Some authors propose combined EUS-ERCP procedures even in children [152, 158, 247]. The use of EUS in the work-up of children with pancreatobiliary pathology may limit exposure to risk of adverse events from ERCP [152]. MRCP and ERCP may be used—the first to document the communication of the cyst with the main pancreatic duct [40, 41, 45, 49, 68, 71, 97, 128, 138, 145, 154, 156, 247, 253, 259, 261] and, the latter, for treating the disease.

*Abscess or intra-abdominal sepsis* occurs in 7–25% of patients with pancreatic injuries; CT-scan or MRI should be performed for diagnosis and to guide treatment [40, 156].

*Pancreatic fistula* occurs in 10–35% of major injuries of the pancreas after operative drainage or resection. A correct diagnosis is very important in planning the treatment. Preoperative cross-sectional imaging and pancreatogram during ERCP are essential. The ERCP, when feasible, is the first step to treat persisting fistulas [11, 40, 41, 48, 49, 61, 71, 156, 233, 262].

The incidence of *post-traumatic pancreatitis* is 17%. Patients with abdominal pain and hyperamylasemia should undergo contrast-enhanced CT-scan for diagnosis wherever possible [40, 156].

#### Post-traumatic exocrine or endocrine function

Although transient post-operative glucose intolerance is common in all critically ill trauma patients, the incidence of persistent new-onset endocrine dysfunction after traumatic distal pancreatectomy is very low (< 4%) [263]; insulin requirement is more frequently associated to proximal pancreatic resections [72, 263] or Whipple procedure [264]. However, both exocrine and endocrine insufficiencies are very rare [4, 10, 15, 16, 45, 52, 54, 58, 69, 265] and no sufficient data exist to have definitive answers and indications [15, 68, 257]. Post-traumatic exocrine or endocrine function in the very long-term seems to be related to overall age and time from injury rather than the surgical treatment [68, 69]. Long-term follow-up is suggested for patients who underwent pancreatic surgery for trauma due to the possibility that the onset of diabetes mellitus may be accelerated by pancreatic resection [53, 264].

## Conclusions

Non-operative management of bilio-duodeno-pancreatic injuries without ductal involvement with or without endoscopic adjuncts is recommended for hemodynamically stable patients. EHBTI can be managed with cholecystectomy for minor injuries, although more severe injuries require surgical reconstruction. Severe bilio-duodeno-pancreatic injuries are rare, often accompanied by hemodynamic instability and may benefit from DCS techniques. Many initial injuries as well as the sequelae of injury may be addressed with percutaneous or endoscopic drainage, and endoscopic stenting. Despite advances in care, morbidity and mortality following severe bilio-duodeno-pancreatic trauma remain high. The management of duodenal, pancreatic, and extrahepatic biliary tree injuries must be multidisciplinary. The management in the initial phase is best accomplished by the trauma or emergency surgeon, and in the reconstructive phase, hepatobiliary surgeons may be helpful and should be consulted.

## Data Availability

Not applicable

## Abbreviations

AAST: American Association for the Surgery for Trauma
BE: Base excess
CT: Computerized tomography
DCS: Damage control surgery
DI: Duodenal injury
DPL: Diagnostic peritoneal lavage
E-FAST: Extended-Focused Assessment with Sonography for Trauma
EHBTI: Extrahepatic biliary tree injury
ERCP: Endoscopic retrograde cholangiopancreatography
EUS: Endoscopic US
fNOM: Failed non-operative management
HIDA: Hepatobiliary scintigraphy
LE: Level of evidence
MRCP: Magnetic resonance cholangiopancreatography
MRI: Magnetic resonance imaging
NGT: Nasogastric tube
NOM: Non-operative management
NPV: Negative predicting value
OIS: Organ injury scale
OM: Operative management
PD: Pancreatic duct
PE: Pyloric exclusion
PI: Pancreatic injury
PPV: Positive predicting value
PTC: Percutaneous transhepatic cholangiogram
SMV: Superior mesenteric vein
TPN: Total parenteral nutrition
US: Ultrasound
WSES: World Society of Emergency Surgery
